# Desmoid fibromatosis of the breast occurring after breast reduction surgery mimicking a carcinoma: A rare case report

**DOI:** 10.1016/j.amsu.2022.103526

**Published:** 2022-04-01

**Authors:** Maryem Bouab, Amjad Harit, Houssine Boufettal, Sakher Mahdaoui, Naima Samouh

**Affiliations:** a Department of Gynecology and Obstetrics, University Hospital Center Ibn Rochd, Casablanca 20100, Morocco; b Faculty of Medicine and Pharmacy, Hassan II University of Casablanca, Casablanca, Morocco

**Keywords:** Desmoid fibromatosis, Breast cancer, Surgical breast reduction, Case report

## Abstract

**Introduction:**

Desmoid tumors are benign mesenchymal tumors developed at the expense of muscular fasciae and aponeuroses. The mammary localization is a rare entity, representing less than 0.2% of all breast tumors. It is characterized by a strictly local evolution and its tendency to recur without giving metastases. Its clinical and radiological presentation is similar to a breast carcinoma which is the main differential diagnosis.

**Case presentation:**

Patient aged 51 years, primigravida primiparous, followed for right breast cancer diagnosed at the age of 49 years for which she received a right mastectomy and axillary lymphnode dissection and contralateral breast reduction. It was a 4 cm infiltrating ductal carcinoma, SBR III Luminal B, 0 N+/20 N with presence of fibrous mastopathy without sign of malignancy at the left breast reduction specimen.The patient received adjuvant chemotherapy, external radiotherapy and hormone therapy.One year after surgery, the patient returned for a four x 2 cm left breast nodule in the upper medial quadrant. The biopsy confirmed the diagnosis of fibromatosis of the breast. A wide local excision was performed.

**Discussion:**

The etiology of this tumor is unknown, however, physical, hormonal and genetic factors play an important role in the development of desmoid tumor.The clinical presentation is similar to breast carcinoma, making it difficult to differentiate this tumor from breast carcinoma. Breast imaging techniques are not specific for desmoid fibromatosis. Treatment is based primarily on complete surgical excision.

**Conclusion:**

Breast fibromatosis is a rare entity, clinically and radiologically mimicking breast cancer. Only histology will provide the diagnosis. The treatment of choice is based on complete surgical excision with healthy safety margins.

## Highlights

• Breast fibromatosis is an uncommon tumor with an uncertain etiology.
• A breast carcinoma is the main differential diagnosis.
• The current report discusses a case of desmoid type fibromatosis of the breast occurring after breast reduction surgery.

## 1 Introduction

Desmoid tumors are benign mesenchymal tumors that develop at the expense of muscular fasciae and aponeuroses. Mammary localization is a rare entity, representing less than 0.2% of all breast tumors [1]. It is characterized by a strictly local evolution and its tendency to recur without giving metastases [2]. Its clinical and radiological presentation is similar to that of a breast carcinoma, which is the main differential diagnosis [3]. Its diagnostic criteria are histological, showing a proliferation of spindle cells (fibro and myofibroblastic without nuclear atypia), arranged in bundles, mixed with bands of collagen, without an epithelial component. Its treatment is surgical and is based on a complete exeresis in order to avoid recurrences. The role of radiotherapy and medical treatments is not clearly defined [4].

We report the case of a patient with a history of breast reduction who presented with breast desmoid fibromatosis on the same breast mimicking a carcinoma.

This work has been reported with respect to the SCARE 2020 criteria [5].

## 2 Case presentation

51-year-old female patient, primigravida primiparous, with a history of spinal trauma at the age of 25 years treated by osteosynthesis, menopausal for 4 years, with no notion of taking oral contraceptives, followed up for right breast cancer diagnosed at the age of 49 years for which she received a right mastectomy and axillary lymphnode dissection and contralateral breast reduction given its volume. It was an infiltrating ductal carcinoma of 4 cm, SBR III Luminal B, 0 N+/20 N with presence of fibrous mastopathy without sign of malignancy at the left breast reduction specimen. The postoperative course was marked by necrosis of the left areolar-nipple plate, a necrosectomy was performed. The metastatic workup was performed at the time of the first presentation of right breast cancer and did not reveal any secondary lesions.The patient was seen in a multi-disciplinary center. She received adjuvant chemotherapy with 6 courses of anthracycline-cyclophosphamide, external radiotherapy with a total dose of 50 Gy, and hormone therapy with tamoxifen citrate.

**Fig. 1:**
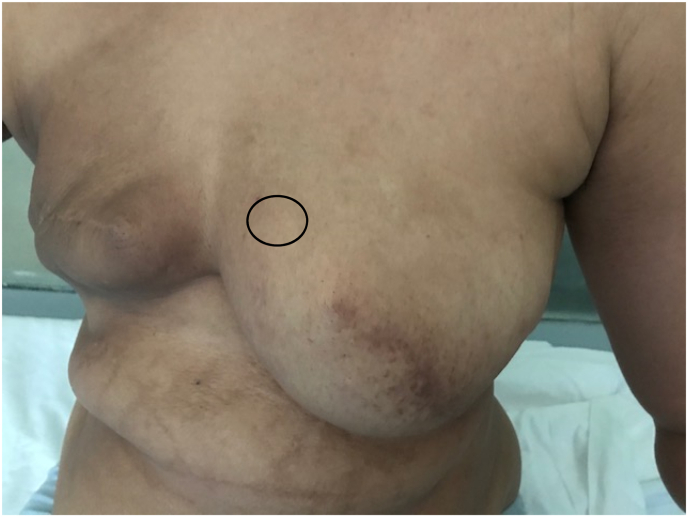
Presence of a 4 × 2 cm nodule in the left superior-internal quadrant, mobile with respect to both planes without opposing inflammatory signs.

**Fig. 2:**
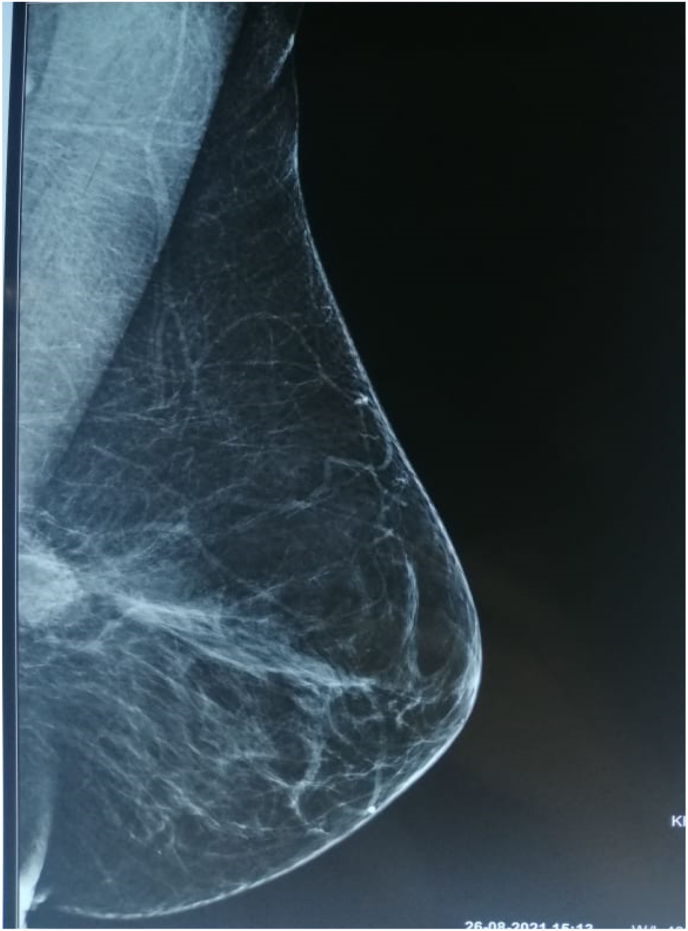
Increased opacity of irregular contour in the superior-internal quadrant of the left breast.

**Fig. 3:**
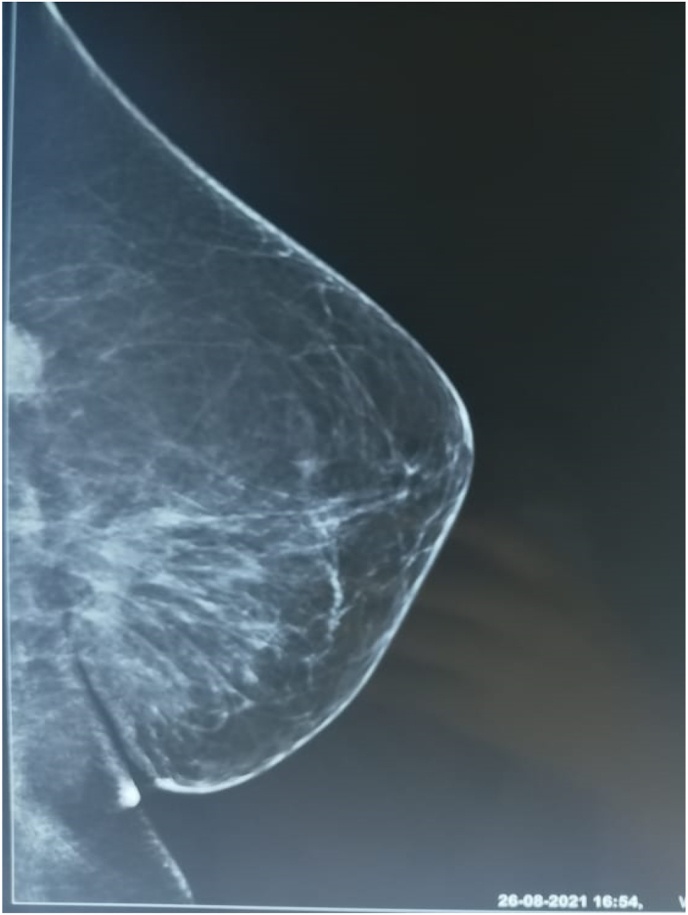
Increased opacity of irregular contour in the superior-internal quadrant of the left breast.

**Fig 4:**
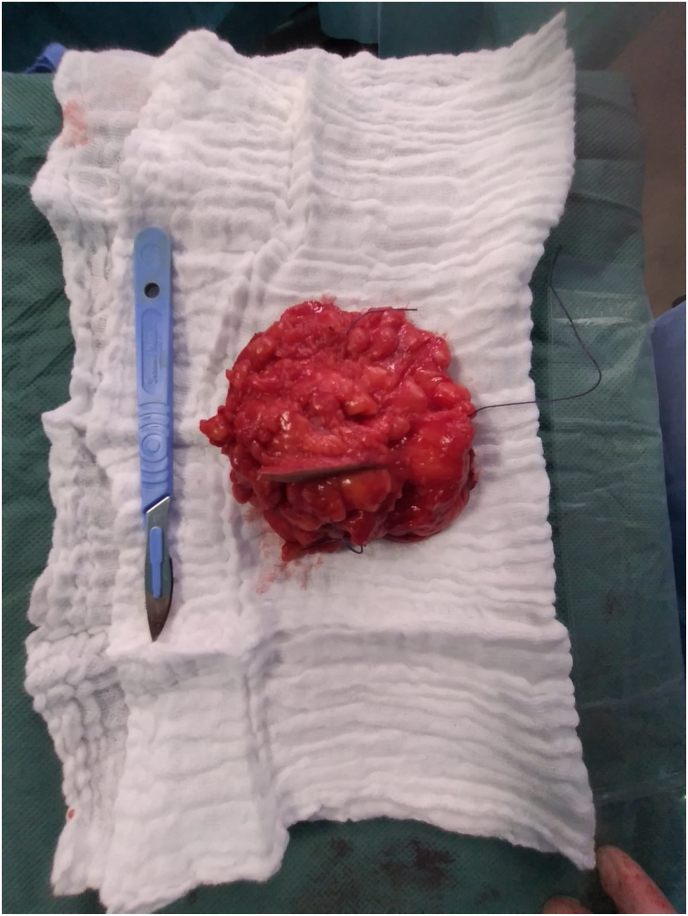
Surgical image of the wide local excision.

One year after the surgery, the patient returned for a nodule of the left breast without inflammatory signs. Clinical examination revealed a four x 2 cm nodule in the upper-internal quadrant mobile in both planes without other associated signs (Fig. 1). Mammography identified a dense, hypoechoic, spiculated, heterogeneous, prepectoral nodule of the left superior-internal quadrant, surrounded by stromal infiltration and responsible for a rupture of the glandular trabeculae, vascularized on color Doppler, measuring approximately 21 × 20 × 12mm classified as Birads 5 of the Amercian College of Radiology ACR. (Fig. 2, Fig. 3). Histological examination of the nodule biopsy showed a spindle cell proliferation with low cellularity, composed of cells with blurred cytoplasmic boundaries and slightly atypical nucleus. The immunohistochemical study showed that the cells expressed beta Catenin diffusely and AML focally. They did not express CD34, desmin or BCL2. This morphological and immunophenotypic appearance was compatible with a desmoid fibromatosis. The patient subsequently underwent a wide local excision (Fig. 4). Anatomopathological examination of the surgical specimen confirmed the presence of a desmoid fibromatosis of 4.3 × 3.2 × 3.1cm with healthy resection limits. Post-operative follow-up was simple. No sign of recurrence was found after 10 months.

## 3 Discussion

The World Health Organization (WHO) defines desmoid fibromatosis as an intermediate soft tissue tumor with the characteristic of deep soft tissue-derived clonal fibroblastic proliferation with the capacity for local infiltration [6].

Desmoid tumors are divided to intra-abdominal and extra-abdominal tumors. The most common site of extra-abdominal desmoid tumors is the extremities [7].

The frequency of breast desmoid tumors reported by various authors does not exceed 0.2% of all breast tumors [1].

Mammary localization is encountered at any age, with a maximum between 20 and 40 years. This breast desmoid fibromatosis can also be seen in men [8].

The pathophysiology is uncertain and could result from several factors, including:

- Initiation of cell proliferation following trauma: Cases have been described after trauma, or after breast surgery such as breast reduction surgery [9] or after breast prosthesis [10].
- A promoting effect of sex hormones: several arguments plead in favour of this effect in addition to the hormonal dependence of desmoid tumors, since fibromatoses occur preferentially in multiparous women, during pregnancy, in a context of hyperoestrogenism or when taking oral contraception [2,11].
- A genetic background due to a disorder in the regulation of fibroblast growth [12].

The clinical presentation is similar to breast carcinoma, making it difficult to differentiate this tumor from breast carcinoma. The degree of presentation of the lesions is variable, the most common being a fixation or retraction of the skin, a hard lump, usually painless unless it compresses the underlying nerve or tissue which recalls the clinical presentation in our patient [13]. On examination it cannot be distinguished from breast carcinoma.

Breast imaging techniques are not specific for desmoid type fibromatosis; it is difficult to distinguish fibromatosis from breast malignancy by imaging [14].

On mammography, desmoids appear as irregular star-shaped tumor, non-calcified, high density masses with speculative margins [15].

Breast Magnetic Resonance Imaging (MRI) can assess the tumor extension of large lesions by showing a poorly defined, spiculated mass, with an iso/T2 hypersignal of variable intensity and heterogeneity [16]. In our patient, breast MRI was not performed given the history of the fracture treated by osteosynthesis.

Histologically, it is a poorly limited lesion, star-shaped, firm and whitish, macroscopically mimicking a carcinoma. Microscopic examination shows a proliferation of spindle cells (fibro and myofibroblastic without nuclear atypia), arranged in clusters, mixed with bands of collagen, without an associated epithelial component [17].

The treatment is essentially based on complete surgical removal. There is no consensus on the safety margin to be respected, which varies according to the studies, from 0.5 cm to 3 cm [4]. Recurrences are frequent (18–29%, 3–6 years), and thoracic muscle and rib involvement is possible.

In some women, a mastectomy is recommended in case of multiple recurrences, in case of a large tumor or in case of difficulty of histological diagnosis [18]. The place of radiotherapy is very controversial in the literature, its effectiveness is dose dependent, and the tumor control is 60–80% for a total dose administered of 50–60 Gy.

The medical treatment is indicated in case of recurrence and contraindication to surgery or radiotherapy. Antiestrogens have shown some efficacy, even in the absence of an estrogen receptor [4].

In our department we opt for surgical treatment with complete excision.

## 4 Conclusion

Breast fibromatosis is a rare entity, clinically and radiologically mimicking breast cancer. Only histology will provide the diagnosis. Complete excision with healthy safety margins is the treatment of choice.

## Ethical approval

I declare on my honor that the ethical approval has been exempted by my establishment.

## Sources of funding

None.

## Author contribution

Bouab Maryem: Corresponding author; writing the paper.

## Trial registry number

None.

## Guarantor

Dr. BOUAB Maryem.

## Provenance and peer review

Not commissioned, externally peer-reviewed.

## Consent

Written informed consent for publication of their clinical details and/or clinical images was obtained from the patient.

## Declaration of competing interest

The authors declare having no conflicts of interest for this article.

## Declaration of competing interest

The authors declare having no conflicts of interest for this article.
